# Atlas of Human Anatomy

**Published:** 1880-01

**Authors:** 


*Atlas of Human Anatomy*, containing 180 large plates arrayed according to Dr. Oesterreicher and Erdl from their original designs from nature and those of the greatest anatomists of modern times, with full and explanatory texts by J. A. Jeancon, M. D. Publishers: A. E. Wilde & Co., Cincinnati, Ohio. This Atlas is published in parts, one of which we have received, and is certainly a commendable publication.

The plates are large, with the organs beautifully portrayed and faithfully drawn, rendering them useful adjuncts in the study of human anatomy.

The publishers deserve great credit for the manner in which they have presented this important work, and the style in which it is executed.

